# Episodes of clinical mastitis and its relationship with duration of treatment and seasonality in crossbred cows maintained in organized dairy farm

**DOI:** 10.14202/vetworld.2016.75-79

**Published:** 2016-01-21

**Authors:** Narender Kumar, A. Manimaran, A. Kumaresan, L. Sreela, Tapas Kumar Patbandha, Shiwani Tiwari, Subhash Chandra

**Affiliations:** 1 Theriogenology Laboratory, Livestock Production Management Section, ICAR - National Dairy Research Institute (NDRI), Karnal - 132 001, Haryana, India; 2 Southern Regional Station, ICAR - National Dairy Research Institute, Adugodi, Hosur road, Bengaluru - 560 030, Karnataka, India; 3 Polytechnic in Animal Husbandry, College of Veterinary Science and A.H., Junagadh Agricultural University, Junagadh - 362 001, Gujarat, India

**Keywords:** clinical mastitis, duration of treatment, episodes, recurrence, relapse, season

## Abstract

**Aim:**

Present study aimed to evaluate the different episodes of clinical mastitis (CM) and influence of duration of treatment and seasonality on the occurrence of different episodes of CM in crossbred cows.

**Materials and Methods:**

A total of 1194 lactation data of crossbred CM cows were collected from mastitis treatment record from 2002 to 2012. Data of CM cows were classified into types of episodes (pattern of repeated or multiple episodes occurrence) and number of episodes (magnitude of multiple cases). Types of episodes were divided as single (clinical cure by a single episode of treatment), relapse (retreatment of the same cow within 21 days), recurrence (new CM at least 21 days after treatment), and both (relapse and recurrence). The season was classified as winter (December to March), summer (April to June), rainy (July to September), and autumn (October to November). The difference between incidences of different types of CM episodes and the association between number or type of CM episodes with duration of treatment and seasons of CM occurrence were analyzed by Chi-square test.

**Results:**

Among 1194 animals suffered with CM, 53, 16, and 18% had the single episode, relapse, and recurrence, respectively; while 13% suffered by both relapse and recurrence. We estimated the duration of treatment and found 80% of the cows treated 1-8 days, in which 65% treated for 1-4 days, while 35% cows were treated for 5-8 days. Further, 12% cows treated for 9-15 days and 7.5% cows treated >15 days. The relationship between duration of treatment and different episodes of CM revealed that 1-8 days treated cows were mostly cured by the single episode with less relapse and recurrence. In contrast, the incidences of recurrence and relapse episodes were higher in cows treated for more than 9 days. The highest incidence of relapse was noticed in winter (36%) than other seasons (10-28%), while the recurrence was less during autumn (9%) compared to other seasons (20-40%).

**Conclusion:**

Cows those suffered by both relapse and recurrence were more susceptible to CM, and they need to be culled from farm to control the transmission of infections. Although the influence of seasonality was difficult to understand, the higher magnitude of relapse and recurrence during winter suggested the adverse effects of cold stress on treatment outcome.

## Introduction

Mastitis continues to be the most frequent and costly disease of dairy animals across the globe. The recent report suggested that the incidence of mastitis has been increased from 20% to 50% in the Indian dairy animals [1]. The environmental and contagious pathogens are the major cause of clinical mastitis (CM), and outcome of clinical cases are depends on the affected cows, mastitis pathogen, treatment, and management including environment related factors [2]. Although the role of genetics background for mastitis susceptibility has been recently suggested, it is believed that mastitis is the management related disease. Hence, prevention through better management has been suggested as a better strategy for controlling the mastitis than therapeutic management. Although, CM has been traditionally considered as single episode disease per lactation, effects of repeated episodes of CM on milk yield, its quality and negative impact on reproduction were suggested by many workers [3-5]. Mastitis is one of the top most reasons for culling and chances of culling or death were increased in repeated or multiple episode cases [5]. Higher productivity of dairy animals over the period of time and its positive correlation with mastitis incidence makes that treatment of CM during lactation is an inevitable tool, and thus it is an important part of economic losses in CM affected dairy animals. However, “successful treatment” of all mastitis cases remains the challenging task for dairy farmers and veterinarians across the globe. Complex nature of disease caused very little progress in understanding the reasons for low cure rate.

The decrease in milk production was observed with repeated episodes CM caused by different organisms, whereas patterns of milk loss varied with both case number and organism [6]. Among the various factors, duration of treatment is believed to be an important determinant of treatment outcome. Although, the minimum recommended duration of treatment against CM was 3 days, the data regarding real practices in organized Indian dairy farm is not available. On other hand, this data would be helpful to calculate the economic losses due to CM treatment in the Indian context. During recent times, it is believed that the production and herd health-related data are very important to understand the efficiency of management in any farm. For instance, observed that changes in milk yield and electrical conductivity as early as 10 days before the actual appearance of clinical diagnosis of any health disorder in dairy cows [7]. Therefore, these data were exploited to use for herd health monitoring programs.

Since mastitis in dairy animals is one of the most important reasons for antibiotic usage, periodical evaluation of mastitis treatment efficacy is an important tool to assess the effectiveness of existing treatment protocol and formulation of effective future treatment strategy in dairy farms. However, mastitis treatment related data were not analyzed as much as production related data. Further, data regarding the overall prevalence of clinical or sub-CM in dairy animals, how many days the CM was treated? How many CM cows in a herd were re-treated? How many cows have multiple mastitis episodes in the single lactation, are important to assess the effectiveness of mastitis treatment protocol [8]. The understandings of various risk factors associated with treatment outcome are important to improve better treatment decision and select the CM cases that are expected to very respond to treatment [9]. However, to our knowledge, no work has been done this area under organized Indian dairy farming conditions.

It is well-known that particular percentage (about 8%) of animals in the farm was responsible for most incidences (about 40%) of CM. Therefore, identification and culling of those most susceptible animals would be very helpful to reduce the transmission of CM in a particular farm. The recent development of Marker Assisted Selections is seemed to be long-term solution for identification of most susceptible animals. On the other hand, where the management and environment are similar to all animals, repeated incidence of CM (more than one episode) in particular cows indirectly suggesting that they could be more susceptible in the farm. Therefore, we quantified the different episodes of CM in organized dairy farm and influence of duration of treatment and seasonality on the occurrence of different episodes of CM in crossbred cows.

## Materials and Methods

### Ethical approval

The present retrospective study was duly approved by the Institutional Animal Ethics Committee (IAEC), ICAR - National Dairy Research Institute, Karnal, Haryana, India.

### Study site

The study was conducted on crossbred cows (Karan Fries) in Livestock Research Centre, ICAR -National Dairy Research Institute, Karnal (Haryana) in the northern part of India. Livestock Research Centre is located at an altitude of 250 m above the mean sea level at 29.42°N latitude and 79.54°E longitude in Eastern Haryana, which comes under the Trans-Gangetic plain agro-climatic zone of India. Meteorological conditions include subtropical weather (hot, humid in summer and near freezing temperature in winter), average annual rainfall about 760-960 mm (received mostly during the months of July and August), and relative humidity 41-85%. The atmospheric temperature varies from near freezing (4°C) during peak winter months at night to about 40°C in summer months during the late afternoon. There are four major seasons that prevail in a year, *viz*., winter (December to March), summer (April to June), rainy (July to September), and autumn (October to November).

### General management with reference to mastitis

The farm animals were maintained under the loose housing system with milking parlors and a well-equipped veterinary health care facility. The nutrient requirements of the animals were mostly met with *ad lib* green fodder, dry fodder, silage, and measured amount of concentrate. The green fodders, grown in the institute farm, were supplied according to the seasonal availability. Three times milking of animals in a day (morning 5.00-6.00 am, noon 12.00-1.00 pm, and in evening 6.00-7.00 pm) using milking machine was the routine practice in the farm. Before milking, pre-stripping and observation of lactating animals for symptoms related to CM by well-trained farm personal and milkers were also a routine procedure in the farm. All the diagnosed animals were immediately treated by a veterinarian as per standard farm practices. Treatment based on personal experience of veterinarians in farm and evaluation through clinical signs and associated physiological changes such as milk yield, feed intake, etc., are the routine follow-up procedure to assess the treatment response in the farm. Mastitis treatment registers were maintained separately in the farm, in which information of animal identification number, quarter affected, symptoms, treatment details, and date of clinical cure were mentioned.

When CM was detected, cleaning and complete removal of milk from affected quarters before administration of antibiotic through intramammary and/or systemic route were routine treatment procedures in the farm. Besides antibiotics, administration of anti-inflammatory, antihistaminic, vitamins, and other preparations as supportive therapy were also routine treatment procedure in farms.

### Data collection, classification, and case definition

The data of CM related information of crossbred cows were collected from mastitis treatment record (n = 1194 lactation with CM) of the institute farm between January 2002 and December 2012. CM was defined as the occurrence of abnormal milk (flakes or chunks in normal or watery milk) with or without swelling of the affected quarter and systemic signs. Clinical cure was defined as the return of quarter and milk secretion to normal, as assessed by visual observation and palpation, at or before milking. Data of CM cows were classified into types of episodes (pattern of repeated or multiple episodes occurrence) and number of episodes (magnitude of multiple cases). Types of episodes were classified as single, relapse, and recurrence. The single episode was defined as CM was clinically cured by the single episode of treatment. Relapse was defined as a retreatment of the same cow within 21 days following an apparent clinical cure. A recurrence was defined as detection of a new CM episode in the same cow 21 days after treatment of the previous episode of CM. Percent higher episodes for multiple or repeated episodes of CM (relapse, recurrence and both) cases were calculated from the incidence of CM (number of animals suffered by CM) and number of CM episodes. Season of CM occurrence was classified as per prevalence in the study site. During classification of the duration of treatment, 1-8 days was kept as minimum due to mastitis caused by potentially invasive pathogens should be treated for 5-8 days [10]. However, the 1-8 days treated cows were divided into 1-4 days and 5-8 days as CM cases mostly treated for 3-5 days.

### Statistical analysis

The frequency of CM incidence in different types of episodes, duration of treatment and seasonality of CM occurrence are represented in percentage of the total incidence. Chi-square test was used to compare the difference between incidences of different types of CM episodes through percent of higher episodes. The association between number or type of CM episodes with duration of treatment and seasons of CM occurrence were also analyzed by Chi-square test through percent calculation. All the analyzes were performed using Sigma Plot 11^®^ software package (Systat software Inc., CA, USA).

## Result and Discussion

The total number of cows suffered with CM during the study period was 1194, and the total episodes of CM were 2277 which is classified into single, relapse, recurrent, and both (relapse and recurrent) CM episodes (Table-1). Among the total animals suffered with CM, 53% of animals (n=638) suffered with single episode, whereas 46% animals (n=556) had multiple episodes (relapse, recurrence, and both relapse or recurrence). The multiple episodes affected animals had 194.78 percent higher episode than total affected animals (i.e. higher episode about 90.7%). We estimated the different types of CM episodes (Table-2) and found 53% of the cows were suffered single episode, 16% cows (n=187) suffered with relapse, and 18% cows (n=214) suffered with recurrence. About 13% of the cows (n=155) were suffered by both relapse and recurrence. Various researchers reported the recurrent episodes of CM [11,12] and suggested as a sensitive indicator of treatment efficacy [13]. The high rates of recurrence may indicate the presence of organisms resistant to the antibiotics used in treatment, and/or poor cure rates [14]. Apparao *et al*. [15] reported the occurrence of recurrent mastitis in quarters defined as cured as well as treatment failures. Recurrent episodes of CM in the same quarter can be caused by persistent intra mammary infection (IMI) or may be associated with recurrent IMI. Bradley and Green [16] reported recurrent episodes of mastitis by the same *Escherichia coli* strain and Bar *et al*. [17] reported 48% to 50% of the third CM episode due to the same pathogen in primiparous and multiparous cows. Very limited duration of immunological memory after each episode of CM could also be the reason for recurrent episodes as it was observed after either vaccination or a full challenge [3,18].

**Table-1:** Incidence of different episodes of CM in crossbred cows.

Episodes	Incidence of CM (%)	No. of CM episodes (%)	% higher episodes
Single	638 (53)	638 (28.02)	0
Multiple	556 (46)	1639 (71.98)	194.78^a^
Total	1194	2277	90.70^b^

Values with different superscript in a column varies significantly at p<0.001, CM=Clinical mastitis

**Table-2:** Incidence of different types of episodes of clinical mastitis in crossbred cows.

Episodes	Incidence of CM (%)	No. of CM episodes (%)	% higher episodes
Single	638 (53)	638 (28.02)	0
Relapse	187 (16)	464 (20.38)	148.12^a^
Recurrence	214 (18)	507 (22.26)	136.91^a^
Both relapse and recurrence	155 (13)	668 (29.34)	330.12^b^
Total	1194	2277	90.70

Values with different superscript in a column varies significantly at p<0.001, CM=Clinical mastitis

We estimated the duration of treatment (Table-3) for clinical cure (2277 mastitis episodes from 1194 cows) and found 80% of the cows (n=961) were treated 1-8 days against 1796 episodes, in which 65% cows (n=620) were treated for 1-4 days against 1146 episodes, while 35% cows (n=341) were treated for 5-8 days against 652 episodes. Further, 12% cows treated for 9-15 days against 305 episodes and 7% cows treated >15 days against 176 episodes. Various researchers reported that treatment of CM for minimum 3 days and extended therapy of 5-8 days for better cure [19]. The duration of treatment in the present study (1-8 days in 80% cows) was higher than that reported in previous studies [20,21]. This difference may be attributable to the omission of severe cases of mastitis in the previous study, different drugs, cow and herd factors, as well as milker or owner awareness of prudent antibiotic usage in lactating animals.

**Table-3:** Relationship between duration of treatment and number of clinical mastitis episodes in crossbred cows.

Duration of treatment (days)	No. of animals treated (%)	No. of CM episodes (%)	% higher episodes
1-8	961 (80.48)	1796 (78.87)	86.02^a^
9-15	144 (12.06)	305 (13.30)	110.41^b^
>15	89 (7.45)	176 (7.72)	97.75^c^
Total	1194	2277	90.70

Values with different superscript in a column varies significantly, 1-8 days varies from 9 to 15 and >15 at p<0.01, 9-15 varies from >15 at p<0.05, CM=Clinical mastitis

We estimated the relationship between duration of treatment and different episodes of CM in cows (Table-4a and -b). Among the 1-8 days treated cows (n=961), we found that the majority of cows were cured by the single episode with fewer episodes of relapse (74%) and recurrence (79%). In contrast, the incidences of recurrence and relapse were higher in cows treated more than nine days duration. It suggests that longer duration of treatment were due to multiple episodes (relapse and recurrence) of CM rather than the longer duration of treatment requirement. On the other hand, these cows may also be more susceptible to mastitis than animals cured by the single episode of treatment. Among the repeatedly suffered cows (n=556), the magnitude of higher episodes was more for those cows suffered by both relapse and recurrence (330%) followed by relapse (148%) and recurrence cows (137%). Collectively, it suggests that cows suffered by both relapse and recurrence were highly susceptible to CM, and they need to be culled from farm to control the transmission of infections.

**Table-4a:** Relationship between duration of treatment and different episodes of clinical mastitis in crossbred cows.

Duration of treatment (days)	Single (%)	Relapse (%)	Recurrence (%)
1-8	528 (82.75)^a^	140 (74.86)^a^	171 (79.90)^a^
9-15	70 (10.97)^b^	28 (14.97)^a^	26 (12.14)^b^
>16	40 (6.26)^b^	19 (10.16)^b^	17 (7.94)^c^
Total	638	187	214

Values with different superscript in a column varies significantly at p<0.05

**Table-4b:** Relationship between 1 and 8 days treatment and different episodes of clinical mastitis in crossbred cows.

Duration of treatment (days)	No. of animals treated (%)	No. of mastitis episodes	% higher episodes	Single	Relapse	Recurrence
1-4	620 (65)	1146	84.83	352^a^	83	104^a^
5-8	341 (35)	652	91.20	176^b^	57	67^b^
Total	961	1798	87.09	528	140	171

Values with different superscript in a column varies significantly; 1-4 varies from 5 to 8 in single at p<0.01; 1-4 varies from 5 to 8 in recurrence at p<0.05

The influence of season on incidence and number of CM episodes is presented in Table-5. The highest incidence of CM was found in winter (36%) and rainy (28%) followed by in summer (25%). The less incidence of CM was noticed in autumn (10%). Similarly, the higher incidence of relapse and recurrence were noticed during winter (36% and 40%, respectively) than other seasons (11-28% and 29-29%, respectively) (Table-6). Despite the average incidence of CM, the incidence of more episodes during summer suggested that treatment efficacy was adversely affected by heat stress. However, overall higher incidence of single or repeated episodes of CM during winter suggested the influence of cold stress-associated impairment of the immunity and thus more relapse of CM.

**Table-5:** Influence of season on incidence and number of clinical mastitis episodes in crossbred cows.

Season	No. of animals treated (%)	No. of mastitis episodes (%)	% higher episodes
Summer	287 (24.03)	578 (25.38)	101.39^a^
Winter	422 (35.34)	828 (36.36)	96.20^b^
Rainy	353 (29.56)	640 (28.11)	81.30^b^
Autumn	132 (11.00)	231 (10.15)	75.01^b^
Total	1194	2277	90.70

Values with different superscript in a column varies significantly at p<0.05

**Table-6:** Influence of season on different episodes of clinical mastitis in crossbred cows.

Season	Single (%)	Relapse (%)	Recurrence (%)
Summer	142 (22.25)^a^	46 (24.59)	61 (28.50)
Winter	208 (32.60)^b^	68 (36.36)	85 (39.71)
Rainy	207 (32.44)^b^	53 (28.34)	49 (22.89)
Autumn	81 (12.69)^c^	20 (10.69)	19 (8.87)
Total	638	187	214

Values with different superscript in a column varies significantly at p<0.05

## Conclusion

Cows those suffered by both relapse and recurrence were more susceptible to CM, and they need to be culled from farm to control the transmission of infections. Although the influence of seasonality was difficult to understand, the higher magnitude of relapse and recurrence during winter suggested the adverse effects of cold stress on treatment outcome. Further, understanding of different episodes of CM with severity, causative agent, and drugs will be really helpful to improve the therapeutic strategies in future.

## Authors’ Contributions

AM and AK designed the study and prepared the manuscript. NK executed while SL, ST, and SC assisted the study, and NK and TKP analyzed the data. All authors read and approved the final manuscript.
